# Notes and Comments

**Published:** 1900-04

**Authors:** 


### Notes and Comments.[1]

SUCCESS.—Mr. George H. Lorimer, editor of the *Saturday Evening Post,* says of success:

"It is hard to learn what success really is; harder to believe that it has its source in copy-book virtues. A great fortune may mean a great failure; a book which sells a hundred thousand copies may represent wasted effort, or worse. Those for whom all that makes life worth living lies within the circumference of the dollar, those to whom Kipling's 'Recessional' was a great poem simply because he could have exchanged it for a check, will not understand this, nor will they ever grasp the real meaning of success. They will fritter their time away in a vain search for their philosopher's stone, or in striking off counterfeits of success.

"But for the man who understands that success is simply doing one thing well, the way is clear, the end sure. Blow by blow, through earth and rock, he will toil till a final stroke shall break through to the crumbling quartz and lay bare the yellow ore of realization. Success lies near for some, for others, deep; let who would find it dig."

---

USE AND ABUSE OF THE BRAIN.—In the course of an address on this subject, Dr. William A. Hammond recently said, "Anxiety causes more brain disorders than any other agency I know of, unless it be love. Many jokes are made about the gray matter of the brain, but I will say, right here, that I have a great respect for the gray matter of the brain. There is no higher organism in all the universe.

---

[1] The assistant editor solicits contributions for this department,—new methods, new remedies and formulas, or any short practical note which may prove of value to the practitioner or student. Address 1338 Walnut Street, Philadelphia.

"It is well for us to know that the emotions cause more unhappiness and crime than any other function of the brain. Human beings are governed by their emotions, and it is well that they should be, though it is the emotions that wear away the brain, and not honest, intellectual work. It is not intellectual work that causes nervous dyspepsia, but the emotions, such as anxiety, fear, sorrow, and love."

Dr. Hammond says he considers that eight hours are sufficient for a man to use his brain, because if he exceeds that time he becomes nervous and fretful, and an exhausted brain is an irritable brain. You may not feel the evil effects of the stress of brain-work at the time, but you will sooner or later, when it is too late. The men that work at night with their brains are the ones that expose themselves to danger and death, which will surely come unless the great strain on the mind is lightened.

---

THE COURTS say dental bills should be paid. Why not? A claim was recently filed and allowed in the Probate Court of Chicago, against the estate of the late George M. Pullman for dental services. The bill was for sixteen hundred dollars, and was presented by Dr. J. N. Crouse. It is reported that there were two charges in the bill of twenty-five dollars each for "time lost," and that the judge, in passing the bill, said, "Well, I suppose Mr. Pullman knew what he was doing when he employed the doctor; the bill is allowed."

---

DEATH TO MOSQUITOES.—*The Public Health Journal* says that it requires two and one-half hours for a mosquito to develop from its first stage, a speck resembling cholera bacteria, to its active and venomous maturity. The insect in all its phases may be instantly killed by contact with minute quantities of permanganate of potassium. It is claimed that one part of this substance in fifteen hundred of solution distributed in mosquito marshes will render the development of larvæ impossible; that a handful of permanganate will oxidize a ten-acre swamp, kill its embryo insects, and keep it free from organic matter for thirty days at a cost of twenty-five cents; that with care a whole State may be kept free of insect pests at a small cost. An efficacious method is to scatter a few crystals widely apart. A single pinch of permanganate has killed all the germs in a thousand-gallon tank.

---

LIQUID AIR AS AN APPETIZER.—The story is reported in the *Bulletin of Pharmacy,* and comes from a Russian physician who placed a dog in a room with the temperature lowered to 100° F. below zero by the use of liquid air. After ten hours the dog was taken out, alive and with an enormous appetite. The physician tried the test himself. After ten hours' confinement in an atmosphere of still, dry cold, his system was intensely stimulated. So much combustion had been required to keep the body warm that an intense appetite was created. The process was continued on the man and the dog, and both grew speedily fat and vigorous; it was like a visit to a bracing northern climate.

---

DR. BOGUE'S METHOD OF TREATING SENSITIVE DENTINE.—In sensitive dentine, when patients are extremely timid, Dr. Bogue dips a pledget of cotton into carbolic acid and then into powdered cocaine, and places it in the cavity. This, he says, will obtund the sensibility enough to use granulated chloride of zinc with little or no pain. In ninety seconds the insensibility of the cavity is complete.—*Dental Brief.*

---

POLISHING FILLINGS.—Keep a cake of calcined magnesia in the cabinet, and when the last disk of cuttlefish is used touch it to the cake and give a brilliant polish to the filling.—*Dental Hints.*

---

THE PERAMBULATING PRESCRIPTION PEDLER is again abroad, offering to sell "office rights," etc. We have seen copies of these prescriptions. The only thing original about them is that they contain dangerous proportions of a dangerous drug. It is a small sort of business for even mean men to engage in, or for even mean men to buy.—*Dominion Dental Journal.*

Refining Gold Scrap.—To those dentists who wish to refine their gold scrap themselves, the following plan is recommended: The scrap should be dissolved in a small quantity of nitro-muriatic acid,—warming hastens the solution; the solution should then be diluted with about three times its volume of water, and nearly neutralized by adding a small quantity of sodium carbonate. The solution should remain slightly acid, or the gold will be precipitated; in that case redissolve by adding a few drops of nitro-muriatic acid. Filter the solution, washing it through with water, then add slowly while stirring a concentrated solution of ferric sulphate, acidulated with a little sulphuric acid. Set the solution aside for twenty-four hours, so that all the gold is precipitated, then decant the liquor through filter-paper to catch any particles of floating gold, wash the precipitate out of the vessel into the filter, palter, roll up the paper, and fuse with plenty of flux.—*Dental Office and Laboratory.*

